# Input of easily available organic C and N stimulates microbial decomposition of soil organic matter in arctic permafrost soil

**DOI:** 10.1016/j.soilbio.2014.04.014

**Published:** 2014-08

**Authors:** Birgit Wild, Jörg Schnecker, Ricardo J. Eloy Alves, Pavel Barsukov, Jiří Bárta, Petr Čapek, Norman Gentsch, Antje Gittel, Georg Guggenberger, Nikolay Lashchinskiy, Robert Mikutta, Olga Rusalimova, Hana Šantrůčková, Olga Shibistova, Tim Urich, Margarete Watzka, Galina Zrazhevskaya, Andreas Richter

**Affiliations:** a University of Vienna, Department of Microbiology and Ecosystem Science, Division of Terrestrial Ecosystem Research, Vienna, Austria; b Austrian Polar Research Institute, Vienna, Austria; c University of Vienna, Department of Ecogenomics and Systems Biology, Division of Archaea Biology and Ecogenomics, Vienna, Austria; d Siberian Branch of the Russian Academy of Sciences, Institute of Soil Science and Agrochemistry, Novosibirsk, Russia; e University of South Bohemia, Department of Ecosystems Biology, České Budějovice, Czech Republic; f Leibniz University Hannover, Institute of Soil Science, Hannover, Germany; g University of Bergen, Centre for Geobiology, Department of Biology, Bergen, Norway; h Siberian Branch of Russian Academy of Sciences, Central Siberian Botanical Garden, Novosibirsk, Russia; i Siberian Branch of Russian Academy of Sciences, VN Sukachev Institute of Forest, Krasnoyarsk, Russia

**Keywords:** Priming, Organic matter decomposition, Phospholipid fatty acid (PLFA), Tundra, Permafrost

## Abstract

Rising temperatures in the Arctic can affect soil organic matter (SOM) decomposition directly and indirectly, by increasing plant primary production and thus the allocation of plant-derived organic compounds into the soil. Such compounds, for example root exudates or decaying fine roots, are easily available for microorganisms, and can alter the decomposition of older SOM (“priming effect”). We here report on a SOM priming experiment in the active layer of a permafrost soil from the central Siberian Arctic, comparing responses of organic topsoil, mineral subsoil, and cryoturbated subsoil material (i.e., poorly decomposed topsoil material subducted into the subsoil by freeze–thaw processes) to additions of ^13^C-labeled glucose, cellulose, a mixture of amino acids, and protein (added at levels corresponding to approximately 1% of soil organic carbon). SOM decomposition in the topsoil was barely affected by higher availability of organic compounds, whereas SOM decomposition in both subsoil horizons responded strongly. In the mineral subsoil, SOM decomposition increased by a factor of two to three after any substrate addition (glucose, cellulose, amino acids, protein), suggesting that the microbial decomposer community was limited in energy to break down more complex components of SOM. In the cryoturbated horizon, SOM decomposition increased by a factor of two after addition of amino acids or protein, but was not significantly affected by glucose or cellulose, indicating nitrogen rather than energy limitation. Since the stimulation of SOM decomposition in cryoturbated material was not connected to microbial growth or to a change in microbial community composition, the additional nitrogen was likely invested in the production of extracellular enzymes required for SOM decomposition. Our findings provide a first mechanistic understanding of priming in permafrost soils and suggest that an increase in the availability of organic carbon or nitrogen, e.g., by increased plant productivity, can change the decomposition of SOM stored in deeper layers of permafrost soils, with possible repercussions on the global climate.

## Highlights

• We tested the response of decomposition in arctic soils to organic matter input.
• Decomposition rates in organic topsoil were hardly affected by substrate additions.
• Decomposition rates in mineral subsoil doubled after addition of organic C.
• Decomposition rates of cryoturbated material doubled after addition of organic N.
• Arctic C stocks might be diminished by increased C and N availability with warming.

## 1 Introduction

Soil organic matter (SOM) decomposition rates in permafrost soils are expected to increase with rising temperatures in the Arctic (Hartley et al., 2008; Conant et al., 2011). In addition to the direct temperature effect, warming might also indirectly affect SOM decomposition, mediated by an increase in plant net primary production. Higher plant productivity is accompanied by an increased input of plant-derived C into the soil (as root litter or root exudates), and can thus increase soil C stocks, as observed for a mineral subsoil in the Alaskan tundra (Sistla et al., 2013). In contrast, higher productivity was found to reduce soil C stocks in a sub-arctic system, offsetting the increase in above- and belowground plant biomass, and, consequently, leading to a net loss of C from the ecosystem (Hartley et al., 2012).

Plants supply the soil microbial community with a range of organic compounds that can be either immediately taken up by microorganisms (e.g., sugars, amino acids and organic acids from root exudation), or that can be easily decomposed (e.g., cellulose and protein from root litter). These organic compounds can stimulate the soil microbial community to decompose more SOM (“priming effect”; Bingeman et al., 1953), (i) by promoting microbial groups that target complex compounds of SOM (Fontaine et al., 2003), (ii) by providing the energy to break down these compounds (Blagodatskaya and Kuzyakov, 2008), or (iii) by providing C for microbial growth, thus increasing microbial N demand and facilitating N mining, i.e., the microbial breakdown of SOM to get access to N (Craine et al., 2007; Dijkstra et al., 2013).

The latter two mechanisms might be of particular importance in arctic soils. Microbial activity in arctic soils is considered N limited (Sistla et al., 2012), suggesting that an increased allocation of plant C to the soil might strongly stimulate N mining. Additionally, microbial activity in subsoil horizons in general is considered energy limited (Fontaine et al., 2007), as the subsoil is poorly rooted, and supply of plant-derived compounds from root exudation and root litter is scarce. With 80% of arctic SOM located below 30 cm (Tarnocai et al., 2009), a large amount of SOM might be protected from decomposition by energy limitation of microbial decomposers, and might thus be particularly susceptible to an increased input of plant-derived compounds. So far, it is unknown how SOM decomposition in different horizons of arctic permafrost soils will respond to an increased input of plant-derived organic compounds, and what mechanisms might be involved.

We here report on the susceptibility of different soil horizons from a tundra ecosystem in the central Siberian Arctic to an increased availability of organic compounds. In a priming experiment, we compared organic topsoil and mineral subsoil material, as well as cryoturbated material, i.e., topsoil material that was buried in the subsoil by freeze–thaw processes (Bockheim, 2007; Tarnocai et al., 2009). Cryoturbated organic matter is common in arctic soils, accounting for approximately 400 Gt of C (Harden et al., 2012). Although it is chemically similar to topsoil organic matter (Xu et al., 2009), it shows retarded decomposition as indicated by low respiration rates (Kaiser et al., 2007) and relatively old radiocarbon ages (Kaiser et al., 2007; Xu et al., 2009; Hugelius et al., 2010). We hypothesized that an increased availability of organic compounds would stimulate SOM decomposition in the subsoil (i.e., in mineral subsoil and cryoturbated horizons) by providing energy for microbial decomposers, but less so in the topsoil, where energy is not limiting. Additionally, we tested if priming of SOM decomposition was connected to N mining, by comparing the effect of organic substrates with and without N. We expected that N-containing substrates would result in a weaker priming effect than substrates without N, by reducing the dependence of the microbial community on SOM as an N source. Finally, we investigated if priming of SOM decomposition was connected to a shift in microbial community composition.

To that end, we analyzed SOM-derived respiration and microbial community composition in soil samples amended with ^13^C-labeled glucose, cellulose, amino acids, or protein, in comparison with unamended controls. We thus compared substrates containing N to substrates without N, as well as monomeric substrates to polymeric substrates. Since monomeric substrates are immediately available for microorganisms, whereas polymeric substrates need to be broken down by extracellular enzymes before microbial uptake, they might differ in their effect on microbial community composition and function, and thus on SOM decomposition (Fontaine et al., 2003).

## 2 Material & methods

### 2.1 Soil sampling

Soils were sampled on the Taymyr peninsula in the central Siberian Arctic (72° 29.57′ N, 101° 38.62′ E), from a shrubby moss tundra (bioclimatic subzone D; CAVM Team, 2003) dominated by *Cassiope tetragona*, *Carex arctisibirica*, *Tomentypnum nitens* and *Aulacomnium turgidum*. The soil was described as a Turbic Cryosol according to the World Reference Base for Soil Resources (IUSS Working Group WRB, 2007) or Typic Aquiturbel according to the US Soil Taxonomy (Soil Survey Staff, 1999), with fine to coarse loamy texture and an active layer depth of around 80 cm at the time of sampling in August 2011. We took samples from three soil horizons in the active layer: We sampled the OA horizon (topsoil material), as well as a buried Ajj (cryoturbated material) and the adjacent BCg horizon (mineral subsoil material), the latter two from a depth of 50–70 cm. Soils were sampled in a 2 m-wide soil profile by pooling samples taken horizontally with a metal soil corer within each horizon. We took care that the mineral subsoil material sampled did not include buried organic material and vice versa. Living roots were carefully removed and samples homogenized by hand directly after sampling. Carbon and nitrogen contents of the individual horizons were 9.4% C and 0.5% N for topsoil material, 4.3% C and 0.2% N for cryoturbated material, and 0.6% C, 0.1% N for mineral subsoil material (determined with a Perkin Elmer 2400 Series II CHNS/O analyzer).

### 2.2 Incubation experiment

To investigate the effect of increased C availability on SOM decomposition, we amended the soils with ^13^C-labeled glucose, amino acids, cellulose, or protein. ^13^C-labeled glucose was purchased from Sigma–Aldrich (U-^13^C, 99 at%), ^13^C-labeled amino acids from Cambridge Isotope Laboratories (algal amino acid mixture, U-^13^C, 97–99 at%), ^13^C-labeled cellulose from Isolife (low degree of polymerization *Cichorium intybus*, U-^13^C, >97 at%), and ^13^C-labeled protein from Sigma–Aldrich (algal crude protein extract, U-^13^C, 98 at%). All substrates were mixed with the respective unlabeled compounds to 10 at% ^13^C before application, and all substrates were applied in dry form.

Aliquots of fresh soil were amended with glucose, amino acids, cellulose or protein of 10 at% ^13^C, in five replicates of 25 g per treatment, or left unamended as controls (three sets of five 25 g replicates). We adjusted the amount of substrate to the approximate C content of each horizon by adding 554 μg C g^−1^ to topsoil material, 138 μg C g^−1^ to cryoturbated material and 55 μg C g^−1^ to mineral subsoil material. One set of controls was immediately harvested to determine the initial state before the start of the incubation; the remaining samples were filled into microcosms (50 ml polypropylene tubes with aeration holes in the bottom, for a detailed description see Inselsbacher et al., 2009) that were then loosely plugged with synthetic wool, and incubated at 10 °C. Since we expected a faster response to monomeric (glucose, amino acids) than to polymeric substrates (cellulose, protein), we incubated samples for either six days (glucose, amino acids, and one set of controls) or ten days (cellulose, protein, and one set of controls).

### 2.3 Respiration

Respiration rates were determined in four replicate microcosms on days 0, 1, 2, 3, 4, 6 (glucose and amino acids, plus controls), or 0, 2, 4, 6, 8, 10 (cellulose and protein, plus controls) after the start of the incubation, with the first sampling immediately after substrate amendment. For respiration measurements, we removed the synthetic wool from the microcosms, attached polypropylene tubes to the microcosms (resulting in an overall headspaces of 133–141 ml) and tightened the systems with rubber seals as described elsewhere (Inselsbacher et al., 2009). We incubated microcosms at 10 °C, took samples of 15 ml from the headspace after 10, 25 and 40 min (5, 10, 20 and 30 min for the glucose treatment), and injected the gas samples into pre-evacuated gas vials. After each sampling, we replaced the removed gas volume with air from a gas bag, filled with ambient air of known CO_2_ concentration and isotopic composition (CO_2_ concentrations of 473–632 ppm depending on the sampling date, 1.09 atom% ^13^C). Concentration and isotopic composition of CO_2_ were measured with a GasBench II system coupled to a Delta V Advantage IRMS (Thermo Scientific), and corrected for the replaced gas volume. We then calculated the contribution of SOM-derived C to respiration using the equation:

(1) $${\text{R}}_{\text{SOM}}={\text{R}}_{\text{total}}\cdot \left(\text{at}{\%}_{{\text{R}}_{\text{total}}}-\text{at}{\%}_{\text{sub}}\right)/\left(\text{at}{\%}_{\text{SOM}}-\text{at}{\%}_{\text{sub}}\right),$$

where R_total_ and R_SOM_ are total and SOM-derived respiration, and $\text{at}{\%}_{{\text{R}}_{\text{total}}}$, at%_SOM_, and at%_sub_ are C isotope compositions (in atom% ^13^C) of total respiration, SOM and the added substrate, respectively.

### 2.4 Microbial biomass and community composition

We estimated microbial biomass as concentration of phospholipid fatty acids (PLFAs), described microbial community composition using PLFAs as biomarkers for different microbial groups, and assessed microbial substrate preferences by tracing C derived from the added substrates into individual PLFAs. Aliquots of 1 g fresh soil were stored in RNAlater (Schnecker et al., 2012), and processed following Frostegård et al. (1991), with the modifications described by Kaiser et al. (2010b). Briefly, PLFAs were extracted from the samples with chloroform/methanol/citric acid buffer, purified on silica columns (LC-Si SPE, Supelco) with chloroform, acetone, and methanol, amended with methyl-nonadecanoate as internal standard, and converted to fatty acid methyl esters (FAMEs) by alkaline methanolysis. We determined concentration and isotopic composition of individual FAMEs on a GC-IRMS system consisting of a Trace GC coupled to a Delta V Advantage IRMS over a GC Isolink interface (Thermo Scientific). Samples were injected in splitless mode at 300 °C, and individual FAMEs were separated on a DB-23 column (Agilent; GC-method: 70 °C for 1.5 min, ramp of 30 °C min^−1^ to 150 °C, 150 °C for 1 min, ramp of 4 °C min^−1^ to 230 °C, 230 °C for 20 min) with 1.5 ml helium min^−1^ as carrier gas. We used qualitative standard mixes (37 Comp. FAME Mix and Bacterial Acid Methyl Esters CP Mix, Sigma–Aldrich) for peak identification, and the internal standard methyl-nonadecanoate for quantification. Concentration and isotopic composition of each PLFA were corrected for C added during derivatization. We further considered only PLFAs that were detectable in all horizons, using i15:0, a15:0, i16:0, i17:0 and a17:0 as markers for Gram-positive bacteria, 16:1ω7, 18:1ω7 and cy17:0(9/10) as markers for Gram-negative bacteria, 18:1ω9 and 18:2ω6 as markers for fungi, and 16:0, 17:0, 18:0, 20:0, 16:1ω5, 16:1ω11 and 19:1ω8 as non-specific PLFAs (Kaiser et al., 2010a, 2010b). We expressed all PLFA values on the basis of C in PLFAs: Total concentrations are presented as total C in all PLFAs, the relative contributions of individual PLFAs as percentages thereof.

To compare the microbial utilization of different substrates across soil horizons, we calculated substrate use efficiency by comparing the amounts of substrate-derived C respired and incorporated into PLFAs, using the equations

(2) $${\text{CR}}_{\text{sub}}={\text{CR}}_{\text{total}}\cdot \left({\text{at\%}}_{{\text{CR}}_{\text{total}}}-{\text{at\%}}_{\text{SOM}}\right)/\left({\text{at\%}}_{\text{sub}}-{\text{at\%}}_{\text{SOM}}\right),$$

(3) $${\text{PLFA}}_{\text{sub}}={\text{PLFA}}_{\text{total}}\cdot \left({\text{at\%}}_{{\text{PLFA}}_{\text{total}}}-{\text{at\%}}_{\text{SOM}}\right)/\left({\text{at\%}}_{\text{sub}}-{\text{at\%}}_{\text{SOM}}\right),$$

and

(4) $$\text{Substrate}\;\text{use}\;\text{efficiency}=\left({\text{PLFA}}_{\text{sub}}\right)/\left({\text{PLFA}}_{\text{sub}}+{\text{CR}}_{\text{sub}}\right)\text{.}$$

CR_total_ and CR_sub_ represent total and substrate-derived C in cumulative respiration, PLFA_total_ and PLFA_sub_ total and substrate-derived C in PLFAs, and ${\text{at\%}}_{{\text{CR}}_{\text{total}}}$, ${\text{at\%}}_{{\text{PLFA}}_{\text{total}}}$, at%_SOM_, and at%_sub_ are C isotope compositions (in atom% ^13^C) of total cumulative respiration, total PLFAs, SOM and the added substrate, respectively. Substrate use efficiency values thus reflect the partitioning of C between respiration and microbial growth (incorporation into PLFAs). Since we did not apply correction factors to convert PLFA concentrations into total microbial C, substrate use efficiency values should only be used for comparison between horizons and treatments.

We additionally determined microbial biomass using chloroform-fumigation-extraction (Kaiser et al., 2011). Samples fumigated with chloroform, as well as unfumigated samples, were extracted with 0.5 M K_2_SO_4_ and analyzed for C concentration with an HPLC-IRMS system in direct injection mode against sucrose standards (for description of the system see Wild et al., 2010). Microbial C was calculated as the difference between fumigated and non-fumigated samples.

### 2.5 Statistical analyses

All statistics were performed with R 2.15.0 (R Development Core Team, 2012), with packages vegan (Oksanen et al., 2012) and ecodist (Goslee and Urban, 2007). We preferentially used parametric methods, and log-transformed data, if necessary, to meet conditions of normal distribution and homoscedasticity. If conditions could not be met, we applied non-parametric methods.

We performed one-way ANOVAs with Tukey's HSD tests between treatments and controls for each soil horizon to test for effects of substrate addition on cumulative SOM-derived respiration, and two-way ANOVA to test for differences in substrate use efficiency across all horizons and substrates. Since PLFA data did not meet the conditions for parametric methods, we applied Mann–Whitney-U tests to assess differences in absolute amounts of total and substrate-derived C, and the relative contributions of individual PLFAs to both. We further performed non-metric multidimensional scaling (NMDS) to describe the effects of substrate addition on microbial community composition (% of total C in PLFAs; scaled data), and to describe the distribution of substrate derived C across the community (% of total substrate-derived C in PLFAs; scaled data). Differences in PLFA patterns were assessed using cluster analysis (Ward hierarchical clustering) and analysis of similarities (ANOSIM). Throughout this text, we use the term “significant” only when referring to statistical results (with differences considered significant at *p* < 0.05).

## 3 Results

### 3.1 Basal respiration and priming effect

**Fig. 1:**
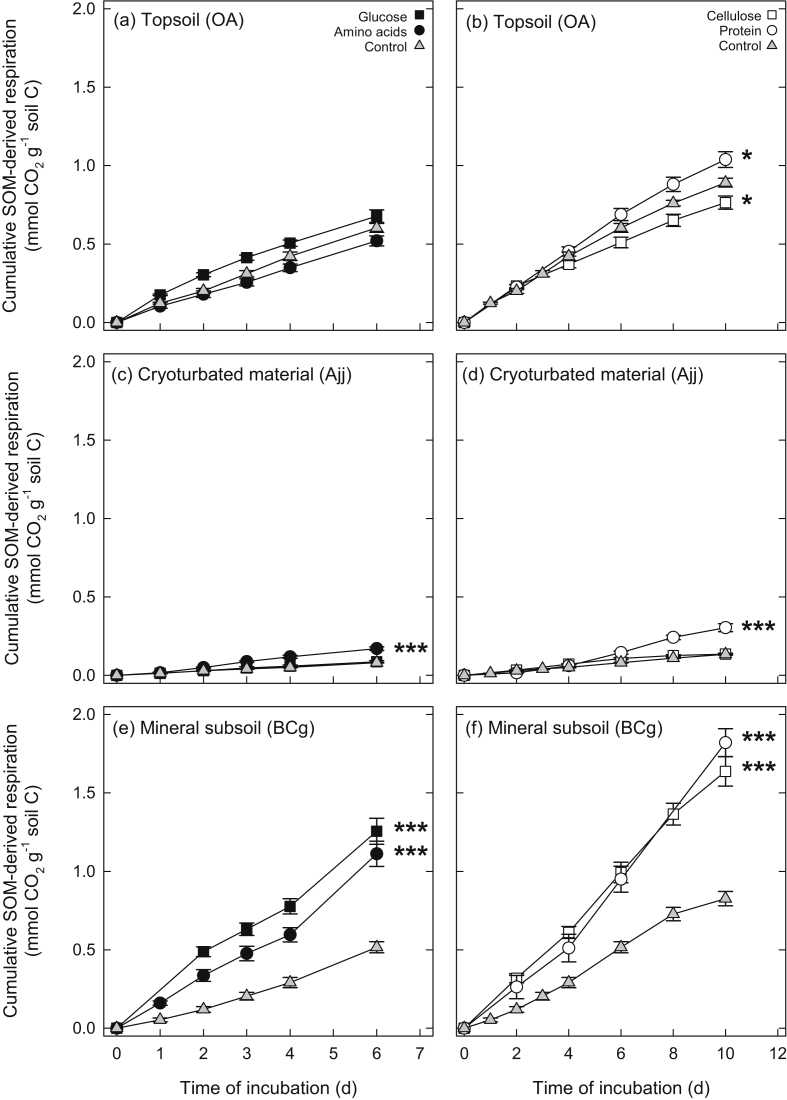
Cumulative SOM-derived respiration of topsoil, cryoturbated, and mineral subsoil horizons from a tundra ecosystem in the central Siberian Arctic. Samples were amended with glucose, amino acids, cellulose or protein (added at levels corresponding to approximately 1% of soil organic C), or left unamended as controls. Points represent means ± standard errors of four replicates. Significant differences between amended samples and controls at the end of the incubation are indicated (*, *p* < 0.05; **, *p* < 0.01; ***, *p* < 0.001).

Respiration rates of unamended control samples differed between soil horizons by more than one order of magnitude, with higher rates in topsoil (0.379 ± 0.030 μmol CO_2_ g^−1^ dry soil h^−1^, mean ± standard error) than in cryoturbated and mineral subsoil horizons (0.024 ± 0.002 and 0.019 ± 0.002 μmol CO_2_ g^−1^ dry soil h^−1^, respectively). Considering the differences in C content between soil horizons, respiration rates were in the same range for topsoil and mineral subsoil (4.050 ± 0.317 and 3.227 ± 0.351 μmol CO_2_ g^−1^ soil C h^−1^), but accounted for only 14% of the topsoil rates in the cryoturbated horizon (0.547 ± 0.043 μmol CO_2_ g^−1^ soil C h^−1^), indicating a slower decomposition of cryoturbated organic matter (Fig. 1, Supplementary Table 1).

**Fig. 2:**
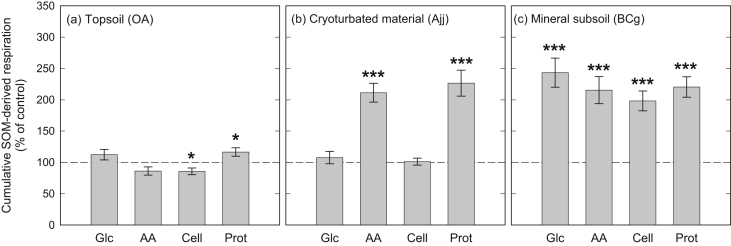
Cumulative SOM-derived respiration of topsoil, cryoturbated, and mineral subsoil horizons from a tundra ecosystem in the central Siberian Arctic, after incubation with glucose (Glc), amino acids (AA), cellulose (Cell) or protein (Prot). Bars represent means ± standard errors of four replicates, expressed as percent of unamended controls which are depicted by the dashed line. Significant differences between amended samples and controls are indicated (*, *p* < 0.05; **, *p* < 0.01; ***, *p* < 0.001).

The addition of organic compounds significantly affected SOM-derived respiration in all horizons. The response of SOM-derived respiration in the topsoil was weak, and significant differences were only observed for cellulose (−14%) and protein treatments (+17%; Fig. 2). In the mineral subsoil, however, all amendments (glucose, amino acids, cellulose, and protein) increased SOM-derived respiration two- to threefold (Fig. 2), demonstrating a strong priming of SOM decomposition by all substrates, which ultimately lead to a higher cumulative respiration than in the topsoil (per soil C; Fig. 1). For cryoturbated material, additions of amino acids and protein doubled SOM-derived respiration, whereas additions of glucose and cellulose had no significant effect (Fig. 2). This suggests that N availability was a major control on SOM decomposition in the cryoturbated horizon.

The addition of organic substrates can increase the turnover of microbial C, resulting in higher respiration rates without changing SOM decomposition (“apparent priming”, Blagodatskaya and Kuzyakov, 2008). In our study, the priming effect in both mineral subsoil (all substrates) and cryoturbated material (amino acids and protein) was in all cases much larger than the initial microbial biomass (Supplementary Table 2), eliminating apparent priming as an explanation for the observed increase in SOM-derived respiration.

### 3.2 Microbial communities

We found different microbial communities in topsoil, cryoturbated, and mineral subsoil horizons, with the latter two harboring the most similar communities. Communities in cryoturbated and mineral subsoil horizons were distinguished from topsoil communities by lower abundances of fungi (18:1ω9, 18:2ω6) and Gram-negative bacteria (18:1ω7, cy17:0), and higher abundances of the non-specific PLFAs 16:0, 18:0 and 20:0 (Supplementary Fig. 1, Supplementary Table 3).

**Fig. 3:**
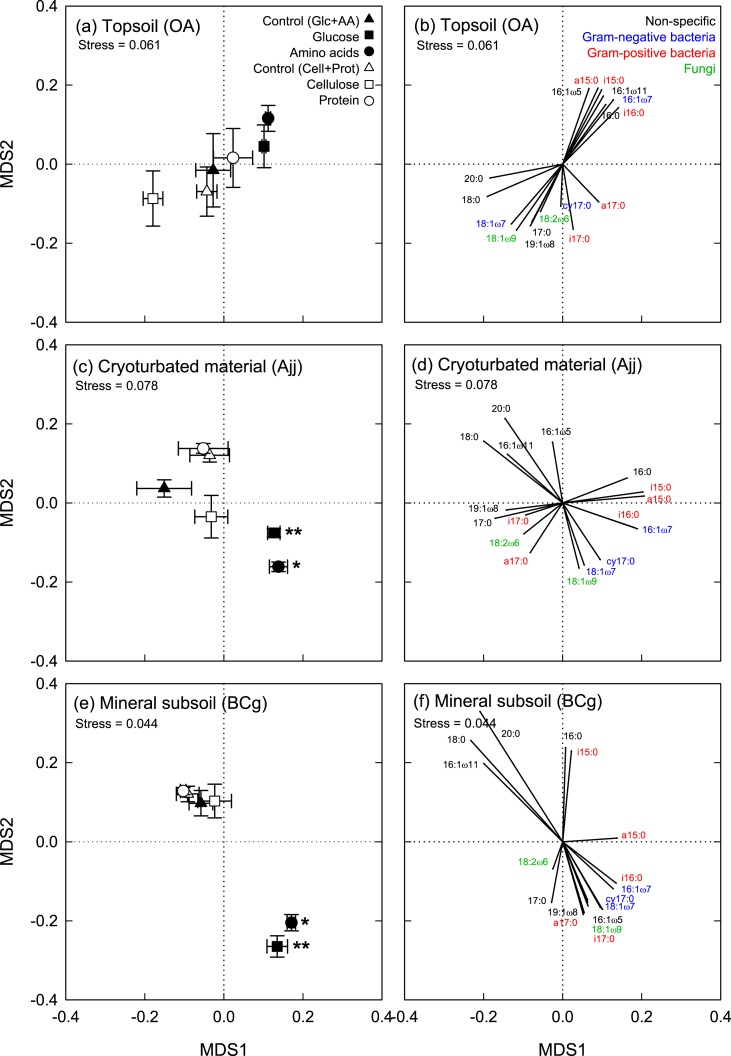
Phospholipid fatty acid (PLFA) patterns in topsoil, cryoturbated, and mineral subsoil horizons from a tundra ecosystem in the central Siberian Arctic. Samples were amended with glucose, amino acids, cellulose or protein, or left unamended as controls. Patterns are displayed by non-metric multidimensional scaling (NMDS) of PLFA abundances (% of total C in PLFAs). Points represent means ± standard errors of five replicates; significant differences to the respective controls are indicated (*, *p* < 0.05; **, *p* < 0.01; ***, *p* < 0.001). Data of PLFA abundances are presented in Supplementary Table 3.

The addition of monomeric substrates (glucose, amino acids) caused a significant increase in the total amount of PLFAs in topsoil and cryoturbated horizons, and a decrease in the mineral subsoil (significant only for glucose; Supplementary Table 3). Microbial community composition in the topsoil was hardly affected by substrate additions, whereas microbial community composition in cryoturbated and mineral subsoil material changed significantly after addition of monomeric substrates (Fig. 3). Specifically, relative abundances of the non-specific PLFAs 18:0 and 20:0 decreased, and abundances of fungi (18:1ω9) and Gram-negative bacteria (cy17:0, 16:1ω7, 18:1ω7) increased (Supplementary Table 3). The addition of monomeric substrates to cryoturbated and mineral subsoil material thus shifted the microbial community composition closer to the topsoil. In contrast, the addition of polymeric substrates (cellulose, protein) had little effect on microbial community composition (Fig. 3, Supplementary Table 3). The community changes in cryoturbated and mineral subsoil material thus suggest a specific stimulation of fast-growing microbial groups by substrates that can be immediately taken up by microorganisms (i.e., glucose and amino acids).

The response of microbial biomass and microbial community composition to substrate additions, did, however, not reflect the response of SOM decomposition. Changes in biomass and community composition were connected to substrate complexity, whereas priming of SOM decomposition was unspecific (mineral subsoil) or connected to N (cryoturbated material).

### 3.3 Substrate use

**Fig. 4:**
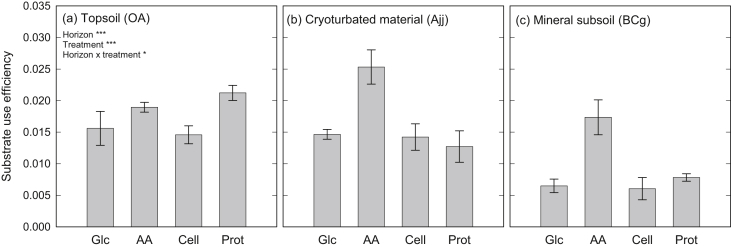
Substrate use efficiency of topsoil, cryoturbated, and mineral subsoil horizons from a tundra ecosystem in the central Siberian Arctic, after incubation with ^13^C-labeled glucose (Glc), amino acids (AA), cellulose (Cell) or protein (Prot). Substrate use efficiency was estimated as the proportion of substrate-derived C in PLFAs over substrate-derived C in both PLFAs and respiration. Bars represent means ± standard errors of four replicates.

Substrate use efficiency was significantly lower in the mineral subsoil than in topsoil and cryoturbated horizons, i.e., a higher proportion of substrate-derived C was respired and a smaller proportion incorporated into PLFAs (Fig. 4). Comparing different substrates, C from amino acids was more efficiently incorporated into PLFAs than C from glucose, cellulose, and protein.

**Fig. 5:**
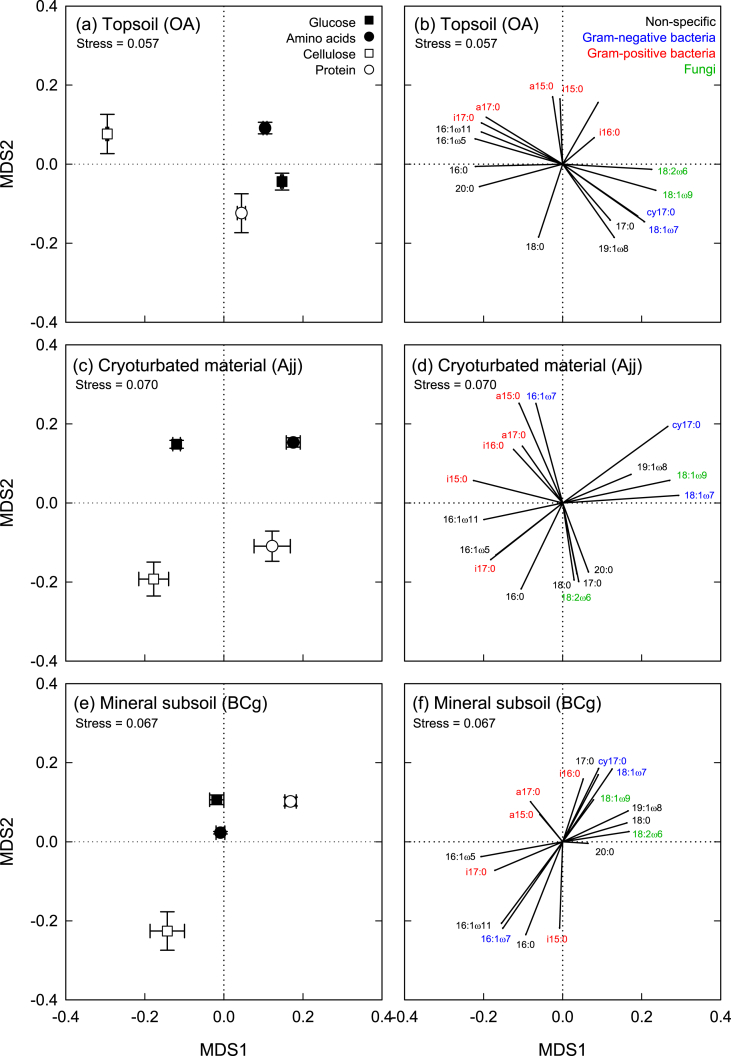
Incorporation patterns of C from glucose, amino acids, cellulose or protein into phospholipid fatty acids (PLFAs), in topsoil, cryoturbated, and mineral subsoil horizons from a tundra ecosystem in the central Siberian Arctic. Patterns are displayed by non-metric multidimensional scaling (NMDS). Points represent means ± standard errors of five replicates; in all horizons, patterns for all substrates were significantly different from each other. Data on the incorporation of substrate-derived C into PLFAs are presented in Supplementary Table 4.

The incorporation of C from different substrates into PLFAs suggests specific substrate preferences of different microbial groups. In cryoturbated material, the incorporation pattern of substrate-derived C depended on the presence of N in the substrate (Fig. 5; separation displayed along MDS 1), and on substrate complexity (separation along MDS 2). In particular, C from N-containing substrates was preferentially incorporated into the Gram-negative biomarkers 18:1ω7, cy17:0, and into the fungal biomarker 18:1ω9, which together contained 47% and 39% of C from amino acids and protein incorporated into PLFAs, but only 25% and 22% of C from glucose and cellulose (Supplementary Table 4). Carbon from monomeric substrates was preferentially incorporated into a15:0 (Gram-positive biomarker) and 16:1ω7 (Gram-negative biomarker), and C from polymeric substrates into 18:2ω6 (fungal biomarker), as well as the non-specific PLFAs 16:0, 17:0, 18:0 and 20:0. A separation due to substrate N content and substrate complexity was not found for other horizons. In topsoil and mineral subsoil, glucose, amino acid and protein treatments had more similar incorporation patterns, whereas cellulose was distinct due to a preferential incorporation into a set of Gram-positive biomarkers (i17:0, a17:0) and non-specific PLFAs (16:0, 16:1ω5, and 16:1ω11) in the topsoil, and into the non-specific PLFA 16:0 in the mineral subsoil (Supplementary Table 4).

## 4 Discussion

Arctic permafrost soils contain large amounts of organic C, with recent estimates of approximately 400 Gt C in mineral subsoil and cryoturbated subsoil horizons each, and 250 Gt C in organic topsoil horizons (Harden et al., 2012). This organic C might be vulnerable to priming, caused by an increasing availability of plant-derived organic compounds with rising temperatures.

We found that organic topsoil, mineral subsoil and cryoturbated horizons responded differently to an increased availability of organic compounds. Substrate additions hardly affected rates of SOM-derived respiration in the organic topsoil, whereas rates more than doubled in the mineral subsoil, irrespective of the substrate added (Fig. 2). Substrate use efficiency (the fraction of substrate taken up invested into PLFAs) was lower in the mineral subsoil than in the other horizons (Fig. 4), indicating that a higher proportion of the available substrate was needed to generate energy. These findings thus support our hypothesis of energy limitation in the mineral subsoil in arctic permafrost soil. Energy limitation of SOM decomposition in mineral subsoil horizons has also been shown for a temperate grassland, where the addition of cellulose induced the mineralization of millennia-old soil organic C (Fontaine et al., 2007).

In our study, priming of SOM decomposition in the mineral subsoil was not connected to an increase in microbial biomass or a change in community composition, since significant community changes were only observed for glucose and amino acid amendments, but not for cellulose or protein (Fig. 3, Supplementary Table 3). Priming was also not connected to N mining of microbial decomposers, since substrates with and without N similarly stimulated SOM decomposition (Fig. 2).

In contrast to the mineral subsoil, SOM decomposition in cryoturbated material was not altered by additions of glucose and cellulose, but doubled when amino acids or protein were added (Fig. 2). This specific response to the N-containing substrates, as well as the higher substrate use efficiency than in the mineral subsoil (Fig. 4), suggest that microbial decomposers in the cryoturbated horizon were not limited in energy, but in N. Cryoturbated material is usually similar in N content and C/N ratio to organic or mineral topsoil horizons, but has lower N transformation rates (Kaiser et al., 2007; Wild et al., 2013), suggesting that N bound in cryoturbated organic material is poorly available for microorganisms (e.g., due to binding to phenolic compounds; Olk et al., 2006). In our study, the C/N ratio of cryoturbated material (21.4) even slightly exceeded the C/N ratio of the organic topsoil (18.5); this might have further promoted N limitation in cryoturbated material.

Carbon from N-containing substrates (amino acids and protein) was preferentially incorporated into fungal (18:1ω9) and Gram-negative bacterial biomarkers (18:1ω7, cy17:0; Fig. 5). Each of these markers was of significantly lower relative abundance in the cryoturbated than in the topsoil horizon, accounting for a total of 13% (cryoturbated) and 40% (topsoil) of C in PLFAs (means of controls; Supplementary Fig. 1). Differences in microbial community composition between topsoil and cryoturbated horizons have previously been suggested to contribute to the slower decomposition of cryoturbated material (Schnecker et al., 2014), in particular the lower abundance of fungi in cryoturbated horizons (Wild et al., 2013; Gittel et al., 2014). Fungi are pivotal for the decomposition of organic material in most terrestrial systems (de Boer et al., 2005; Strickland and Rousk, 2010), and can benefit from higher N availability (Rousk and Bååth, 2007; Grman and Robinson, 2013; Koranda et al., 2014). However, although the preference of specific microbial groups for N-containing compounds is striking, this does not necessarily identify these groups as responsible for priming.

Nitrogen addition often decreases SOM decomposition rates, by reducing N mining of microbial decomposers (e.g., Craine et al., 2007). On the other hand, in systems of strong N limitation, where low N availability limits the production of SOM-degrading enzymes, N addition can stimulate decomposition by facilitating enzyme production by fungi (Allison et al., 2009). Since in our study the amendment of amino acids and protein to cryoturbated material did not systematically increase microbial biomass or the abundance of specific microbial groups (Fig. 3, Supplementary Table 3), we suggest that microorganisms invested the additional N in the production of extracellular enzymes.

Within a relatively short period, SOM decomposition in the subsoil, but not in the topsoil, was strongly stimulated by increased availability of organic C (mineral subsoil) or organic N (cryoturbated material). Rising temperatures in the Arctic (IPCC, 2007) are expected to promote soil C availability by increasing plant primary productivity (Bhatt et al., 2010; Xu et al., 2013) and thus C allocation from plants to the soil. Nitrogen availability might also benefit from higher plant productivity, if the additional input of N-containing compounds by plants (e.g., proteins) exceeds the increase in plant N uptake (Weintraub and Schimel, 2005). In addition, N availability might further increase if SOM decomposition rates increase (Nadelhoffer et al., 1991; Hobbie, 1996; Schimel et al., 2004; Schaeffer et al., 2013).

In our study, mineral subsoil material was particularly susceptible to priming, with rates of SOM-derived respiration exceeding topsoil rates (as related to soil C) after substrate addition. Our findings are thus in contrast to observations of increased C storage in mineral subsoil horizons with warming (Sistla et al., 2013), but support predictions of high C losses from arctic soils if plant productivity increases (Hartley et al., 2012). These contrasting findings on the impact of higher plant productivity on soil C stocks point to a strong context-dependency of priming effects in arctic soils. Our study provides key insights into the mechanisms behind priming effects in permafrost soils; this will allow possible feedbacks from belowground plant–microbe interactions to be incorporated into larger-scale models of arctic C balances in a future climate.
