# Supplementary material for: Input of easily available organic C and N stimulates microbial decomposition of soil organic matter in arctic permafrost soil
Source: Soil Biol Biochem. 2014 Aug;75(100):143–51. doi: 10.1016/j.soilbio.2014.04.014 (PMC4064687; doi:10.1016/j.soilbio.2014.04.014)
Supplement: Supplementary file 1 [file mmc1.pdf]

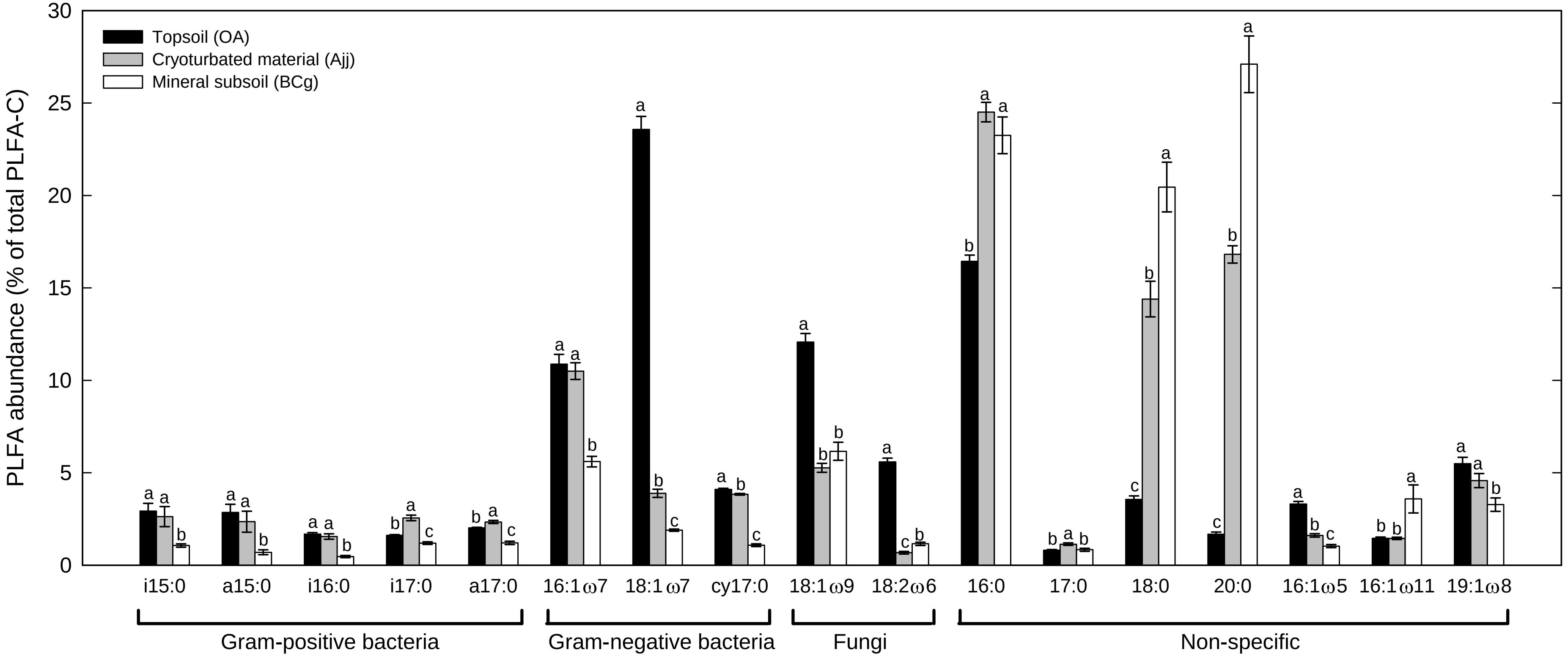

Supplementary Fig. 1. Relative abundances of individual phospholipid fatty acids (PLFAs) in control samples of topsoil, cryoturbated, and mineral subsoil horizons from a tundra ecosystem in the central Siberian Arctic. Bars represent means ± standard errors of ten replicates (six and ten day controls combined), different letters indicate significant differences between soil horizons ($p < 0.05$).

Supplementary Table 1. Respiration rates of unamended control samples of topsoil, cryoturbated, and mineral subsoil material from a tundra ecosystem in the central Siberian Arctic. Values are means of all time points, with standard errors in brackets.

	Respiration per dry soil µmol CO_2 g^{-1} dry soil h^{-1}	Respiration per soil C µmol CO_2 g^{-1} soil C h^{-1}
Topsoil	0.379 (0.030)	4.050 (0.317)
Cryoturbated material	0.024 (0.002)	0.547 (0.043)
Mineral subsoil	0.019 (0.002)	3.227 (0.351)

Supplementary Table 2. Comparison between initial microbial biomass (determined by chloroform-fumigation-extraction) and the priming effect after addition of glucose, amino acids, cellulose or protein to topsoil, cryoturbated, and mineral subsoil material from a tundra ecosystem in the central Siberian Arctic. Priming effect is given as the difference in cumulative SOM-derived respiration between amended samples and unamended controls after six days (glucose and amino acids) or ten days (cellulose and protein) of incubation. Values are means of five (initial biomass) or four (priming effect) replicates, with standard errors in brackets. Bold values indicate a significant difference in SOM-derived respiration between amended samples and controls at $p < 0.05$.

		Topsoil<br>µmol C g^{-1} soil C	Cryoturbated material<br>µmol C g^{-1} soil C	Mineral subsoil<br>µmol C g^{-1} soil C
Initial biomass		504.42 (44.09)	33.61 (8.25)	72.25 (20.53)
Priming effect	Glucose	74.49 (48.60)	6.15 (7.94)	**739.63 (90.23)**
	Amino acids	-83.25 (42.06)	**89.90 (11.43)**	**595.85 (88.11)**
	Cellulose	**-127.29 (50.16)**	1.63 (7.37)	**811.25 (104.32)**
	Protein	**147.12 (57.63)**	**169.65 (25.75)**	**994.56 (100.14)**

Supplementary Table 3. Total amount of phospholipid fatty acids (PLFAs; in µmol C g^{-1} soil C), and relative abundances of individual PLFAs (in % of total C in PLFAs), for topsoil, cryoturbated, and mineral subsoil horizons from a tundra ecosystem in the central Siberian Arctic. Samples were amended with glucose (Glc), amino acids (AA), cellulose (Cell) or protein (Prot), or left unamended (controls), and incubated for six days (glucose, amino acids and control Glc+AA) or ten days (cellulose, protein and control Cell+Prot). Values are means of five replicates, with standard errors in brackets. Bold values indicate a significant difference to the respective control at $p < 0.05$.

	Topsoil						Cryoturbated material						Mineral subsoil					
	Control Glc+AA	Glc	AA	Control Cell+Prot	Cell	Prot	Control Glc+AA	Glc	AA	Control Cell+Prot	Cell	Prot	Control Glc+AA	Glc	AA	Control Cell+Prot	Cell	Prot
Total	89.9 (8.0)	**122.2 (3.1)**	**131.3 (8.2)**	79.7 (2.8)	70.9 (4.8)	92.1 (9.7)	21.5 (3.5)	**37.8 (2.0)**	**39.0 (2.2)**	29.0 (2.4)	26.4 (1.5)	24.8 (2.3)	49.5 (5.7)	**28.3 (2.9)**	40.5 (2.3)	73.3 (4.7)	59.3 (7.1)	57.9 (2.8)
i15:0	3.3 (0.7)	3.9 (0.4)	4.3 (0.4)	2.6 (0.5)	2.4 (0.7)	3.5 (0.7)	2.0 (0.8)	**5.7 (0.6)**	4.3 (0.5)	3.4 (0.6)	3.4 (0.7)	2.7 (0.7)	1.1 (0.2)	0.8 (0.2)	1.3 (0.2)	1.0 (0.1)	1.4 (0.2)	1.1 (0.1)
a15:0	3.2 (0.7)	3.7 (0.5)	4.2 (0.4)	2.5 (0.5)	2.3 (0.8)	3.4 (0.8)	1.7 (0.8)	**5.8 (0.6)**	4.5 (0.6)	3.2 (0.6)	3.2 (0.8)	2.4 (0.7)	0.8 (0.2)	1.2 (0.3)	2.0 (0.3)	0.6 (0.1)	1.0 (0.2)	0.5 (0.0)
i16:0	1.7 (0.1)	2.2 (0.1)	**2.2 (0.1)**	1.6 (0.1)	1.4 (0.2)	1.8 (0.1)	1.4 (0.2)	**3.1 (0.1)**	**2.6 (0.1)**	1.8 (0.1)	2.1 (0.1)	1.4 (0.2)	0.6 (0.1)	**1.1 (0.1)**	**1.1 (0.1)**	0.4 (0.1)	0.7 (0.2)	0.3 (0.0)
i17:0	1.6 (0.1)	1.7 (0.1)	1.6 (0.0)	1.6 (0.0)	1.6 (0.1)	1.6 (0.1)	2.5 (0.1)	2.7 (0.1)	2.7 (0.1)	2.6 (0.3)	2.5 (0.1)	2.7 (0.2)	1.2 (0.1)	**5.0 (0.8)**	**5.5 (1.2)**	1.2 (0.1)	1.8 (0.5)	0.9 (0.2)
a17:0	2.0 (0.1)	**2.6 (0.3)**	2.1 (0.1)	2.1 (0.0)	2.0 (0.1)	2.1 (0.1)	2.4 (0.1)	2.7 (0.1)	2.5 (0.1)	2.2 (0.1)	2.5 (0.2)	2.2 (0.2)	1.4 (0.1)	**2.8 (0.2)**	**2.4 (0.2)**	1.1 (0.1)	1.7 (0.4)	1.1 (0.1)
16:1ω7	11.1 (0.9)	12.7 (0.4)	**13.9 (0.4)**	10.7 (0.6)	10.2 (0.8)	11.9 (0.7)	10.3 (0.8)	**15.2 (0.2)**	**16.7 (0.4)**	10.8 (0.4)	12.2 (0.6)	12.1 (0.9)	6.0 (0.5)	**11.1 (1.0)**	**14.2 (0.5)**	5.2 (0.3)	6.8 (0.9)	5.3 (0.3)
18:1ω7	23.0 (1.1)	**20.7 (0.5)**	21.0 (0.3)	24.2 (0.9)	23.5 (0.9)	23.2 (1.3)	4.2 (0.3)	**5.3 (0.2)**	**7.4 (0.2)**	3.5 (0.1)	4.6 (0.5)	3.7 (0.2)	1.9 (0.1)	**6.2 (0.3)**	**7.0 (0.4)**	1.9 (0.1)	**2.6 (0.3)**	2.3 (0.2)
cy17:0	4.0 (0.0)	4.2 (0.1)	4.1 (0.1)	4.2 (0.1)	4.1 (0.1)	3.8 (0.1)	3.9 (0.1)	**5.1 (0.1)**	**6.0 (0.1)**	3.8 (0.1)	3.9 (0.2)	**3.2 (0.1)**	1.3 (0.1)	**3.7 (0.1)**	**3.5 (0.2)**	0.9 (0.0)	**1.4 (0.1)**	**1.1 (0.0)**
18:1ω9	11.7 (0.8)	11.5 (0.2)	11.0 (0.1)	12.5 (0.5)	12.4 (0.6)	11.9 (0.6)	5.7 (0.3)	**7.2 (0.2)**	**10.6 (0.4)**	4.8 (0.3)	**8.2 (0.9)**	4.2 (0.2)	5.5 (0.4)	**11.3 (0.2)**	**11.0 (0.7)**	6.8 (0.8)	8.7 (0.7)	5.6 (0.4)
18:2ω6	5.6 (0.3)	5.8 (0.2)	5.6 (0.2)	5.6 (0.3)	5.8 (0.2)	5.2 (0.2)	0.8 (0.1)	0.6 (0.0)	0.6 (0.1)	0.5 (0.0)	**0.8 (0.1)**	0.4 (0.1)	1.3 (0.1)	1.4 (0.1)	1.7 (0.6)	1.0 (0.1)	1.2 (0.4)	0.9 (0.1)
16:0	16.6 (0.6)	17.6 (0.2)	17.3 (0.2)	16.2 (0.4)	16.2 (0.5)	16.6 (0.6)	23.7 (0.6)	**27.0 (0.4)**	24.5 (0.2)	25.5 (0.6)	24.7 (0.5)	24.4 (0.7)	24.2 (1.9)	19.9 (1.3)	21.2 (1.1)	22.3 (0.7)	22.9 (2.1)	20.6 (0.7)
17:0	0.8 (0.1)	**0.7 (0.0)**	0.7 (0.0)	0.8 (0.0)	0.7 (0.0)	0.8 (0.0)	1.2 (0.1)	**1.0 (0.1)**	**0.9 (0.1)**	1.0 (0.1)	1.3 (0.1)	1.0 (0.1)	0.9 (0.2)	1.2 (0.1)	1.0 (0.1)	0.7 (0.1)	1.0 (0.2)	0.9 (0.0)
18:0	3.6 (0.4)	**2.8 (0.1)**	**2.3 (0.1)**	3.5 (0.2)	4.5 (0.5)	3.1 (0.3)	15.5 (1.4)	**7.1 (0.2)**	**6.0 (0.3)**	13.0 (1.1)	10.7 (0.7)	13.9 (1.4)	22.3 (2.2)	**10.7 (0.5)**	**10.4 (1.5)**	18.6 (1.3)	18.6 (2.4)	21.3 (0.6)
20:0	1.8 (0.2)	1.1 (0.1)	**0.9 (0.0)**	1.5 (0.1)	**4.5 (0.2)**	1.2 (0.1)	17.2 (0.8)	**4.4 (0.2)**	**4.2 (0.3)**	16.4 (0.4)	11.1 (2.2)	17.8 (0.5)	24.1 (1.1)	**3.5 (0.3)**	**2.5 (0.3)**	30.1 (2.2)	23.7 (0.9)	30.7 (0.6)
16:1ω5	3.3 (0.2)	3.5 (0.1)	3.4 (0.1)	3.3 (0.2)	3.3 (0.2)	3.4 (0.1)	1.5 (0.1)	1.5 (0.0)	0.9 (0.0)	1.3 (0.0)	1.2 (0.1)	1.2 (0.1)	1.0 (0.1)	**2.8 (0.5)**	**2.1 (0.2)**	1.1 (0.2)	1.4 (0.3)	0.8 (0.1)
16:1ω11	1.5 (0.1)	1.6 (0.0)	1.6 (0.0)	1.5 (0.1)	1.4 (0.1)	1.5 (0.1)	1.6 (0.1)	**1.2 (0.0)**	**0.9 (0.0)**	1.3 (0.0)	1.2 (0.1)	1.2 (0.1)	2.9 (0.6)	**1.4 (0.1)**	**1.4 (0.2)**	4.2 (1.4)	2.7 (0.2)	3.5 (0.2)
19:1ω8	5.2 (0.6)	4.1 (0.3)	**3.3 (0.1)**	5.8 (0.4)	**4.4 (0.3)**	5.0 (0.4)	4.6 (0.5)	4.5 (0.1)	4.1 (0.2)	4.5 (0.7)	6.1 (0.9)	5.0 (0.5)	3.6 (0.7)	**15.9 (1.8)**	**10.3 (0.3)**	3.0 (0.1)	3.4 (0.7)	3.3 (0.2)

Supplementary Table 4. Total amount of substrate-derived C in phospholipid fatty acids (PLFAs; in µmol C g^{-1} soil C), and incorporation into individual PLFAs (in % of total substrate-derived C in PLFAs), for topsoil, cryoturbated, and mineral subsoil horizons from a tundra ecosystem in the central Siberian Arctic. Samples were amended with glucose (Glc), amino acids (AA), cellulose (Cell) or protein (Prot), and incubated for six days (glucose and amino acids) or ten days (cellulose and protein). Values are means of five replicates, with standard errors in brackets. Different letters indicate significant differences between substrates at $p < 0.05$.

	Topsoil								Cryoturbated material								Mineral subsoil							
	Glc	AA	Cell	Prot	Glc	AA	Cell	Prot	Glc	AA	Cell	Prot	Glc	AA	Cell	Prot	Glc	AA	Cell	Prot	Glc	AA	Cell	Prot
Total	7.6 (2.4)	7.5 (0.8)	0.4 (0.1)	1.8 (0.5)					1.7 (0.2)	3.3 (2.0)	0.0 (0.0)	0.2 (0.1)					2.2 (0.8)	3.4 (0.9)	0.0 (0.0)	0.5 (0.0)				
i15:0	2.6 (0.3)	3.8 (0.3)	3.4 (0.9)	3.1 (0.6)	b	a	ab	ab	4.6 (0.4)	1.9 (0.2)	4.5 (1.1)	2.7 (0.7)	a	b	a	ab	0.6 (0.2)	0.7 (0.2)	1.8 (0.7)	0.8 (0.2)	a	a	a	a
a15:0	2.6 (0.4)	2.9 (0.3)	3.2 (0.9)	2.3 (0.5)	a	a	a	a	8.1 (0.6)	4.2 (0.4)	2.9 (0.7)	2.2 (0.6)	a	b	bc	c	3.9 (1.0)	4.2 (0.8)	0.6 (0.3)	1.0 (0.2)	a	a	b	b
i16:0	2.1 (0.1)	2.0 (0.1)	1.8 (0.3)	1.7 (0.1)	a	ab	ab	b	4.0 (0.0)	0.6 (0.0)	1.2 (0.1)	1.5 (0.2)	a	c	b	b	2.0 (0.1)	0.6 (0.0)	n.d.	1.3 (0.0)	a	c		b
i17:0	1.0 (0.0)	1.7 (0.1)	2.8 (0.1)	1.0 (0.1)	c	b	a	c	1.7 (0.0)	0.9 (0.0)	3.1 (0.2)	1.1 (0.2)	b	c	a	c	0.8 (0.0)	0.8 (0.1)	2.1 (1.0)	0.6 (0.1)	a	a	a	a
a17:0	1.2 (0.1)	2.3 (0.1)	3.1 (0.1)	1.5 (0.1)	d	b	a	c	2.3 (0.1)	0.7 (0.0)	0.9 (0.1)	1.0 (0.1)	a	c	bc	b	3.5 (0.3)	1.7 (0.2)	0.9 (0.5)	0.8 (0.1)	a	b	bc	c
16:1ω7	15.0 (0.8)	23.0 (1.0)	15.6 (0.8)	15.3 (1.1)	b	a	b	b	31.7 (0.4)	26.8 (0.8)	23.7 (0.7)	25.5 (2.2)	a	b	c	bc	28.7 (2.3)	31.2 (1.6)	42.6 (4.7)	15.2 (1.0)	a	a	a	b
18:1ω7	26.7 (1.1)	26.5 (0.7)	15.7 (1.9)	30.8 (2.3)	a	a	b	a	14.4 (0.7)	18.9 (0.6)	11.6 (1.4)	20.8 (1.7)	b	a	b	a	18.2 (1.7)	15.4 (0.7)	6.0 (2.0)	21.7 (1.0)	ab	b	c	a
cy17:0	3.3 (0.1)	3.5 (0.2)	2.1 (0.1)	4.4 (0.2)	b	b	c	a	5.2 (0.1)	9.2 (0.3)	2.5 (0.4)	6.1 (0.3)	c	a	d	b	7.2 (0.3)	5.0 (0.2)	0.5 (0.2)	6.0 (0.1)	a	c	d	b
18:1ω9	14.8 (0.4)	9.9 (0.2)	4.4 (0.6)	8.5 (0.6)	a	b	c	b	5.2 (0.2)	18.8 (1.0)	8.2 (0.9)	11.7 (1.0)	c	a	b	b	11.1 (1.1)	22.2 (2.3)	6.9)2.5)	18.3 (0.7)	b	a	b	a
18:2ω6	6.2 (0.2)	3.2 (0.2)	0.4 (0.0)	1.9 (0.3)	a	b	d	c	0.2 (0.0)	n.d.	1.4 (0.2)	2.4 (0.2)	c		b	a	0.2 (0.1)	0.0 (0.0)	0.4 (0.3)	5.0 (0.3)	b	c	bc	a
16:0	17.2 (0.1)	16.3 (0.1)	25.5 (0.4)	21.2 (1.0)	c	d	a	b	18.2 (0.2)	15.8 (0.2)	24.2 (1.8)	19.5 (0.2)	b	c	a	a	19.5 (0.5)	15.3 (0.4)	31.0 (2.5)	20.6 (0.7)	b	c	a	b
17:0	0.5 (0.0)	0.5 (0.0)	0.3 (0.0)	1.0 (0.1)	b	b	c	a	0.6 (0.0)	0.4 (0.0)	0.7 (0.1)	0.7 (0.1)	a	b	a	ab	1.2 (0.2)	0.8 (0.1)	0.2 (0.1)	1.3 (0.2)	ab	b	c	a
18:0	1.5 (0.0)	0.8 (0.0)	1.6 (0.2)	1.9 (0.2)	a	b	a	a	1.1 (0.1)	0.4 (0.0)	2.1 (0.6)	2.8 (0.3)	b	c	abc	a	1.0 (0.1)	0.7 (0.1)	0.9 (0.3)	5.0 (0.5)	b	c	bc	a
20:0	0.1 (0.0)	0.0 (0.0)	0.4 (0.1)	0.2 (0.2)	b	b	a	a	0.2 (0.0)	0.0 (0.0)	0.3 (0.1)	0.6 (0.1)	b	c	ab	a	0.0 (0.0)	0.0 (0.0)	0.3 (0.2)	0.7 (0.0)	b	b	ab	a
16:1ω5	2.2 (0.1)	1.7 (0.1)	16.6 (2.3)	1.9 (0.2)	b	c	a	bc	1.4 (0.0)	0.8 (0.0)	11.7 (1.5)	0.8 (0.0)	b	c	a	c	1.2 (0.1)	1.0 (0.1)	1.6 (0.8)	0.2 (0.1)	a	a	ab	b
16:1ω11	1.0 (0.0)	0.8 (0.0)	2.2 (0.2)	0.9 (0.1)	b	c	a	bc	0.7 (0.0)	0.3 (0.0)	1.2 (0.2)	0.1 (0.0)	b	c	a	d	0.5 (0.0)	0.3 (0.0)	4.3 (1.3)	0.2 (0.1)	a	b	ab	ab
19:1ω8	2.1 (0.2)	1.1 (0.1)	1.0 (0.1)	2.6 (0.4)	a	b	b	a	0.5 (0.1)	0.3 (0.0)	0.0 (0.0)	0.6 (0.1)	a	a	b	a	0.3 (0.1)	0.2 (0.1)	n.d.	1.3 (0.2)	b	b		a
